# The impact of TLR2 and aging on the humoral immune response to *Staphylococcus aureus* bacteremia in mice

**DOI:** 10.1038/s41598-023-35970-3

**Published:** 2023-05-31

**Authors:** Priti Gupta, Zhicheng Hu, Pradeep Kumar Kopparapu, Meghshree Deshmukh, Tibor Sághy, Majd Mohammad, Tao Jin, Cecilia Engdahl

**Affiliations:** 1 Department of Rheumatology and Inflammation Research, Institute of Medicine, Sahlgrenska Academy, University of Gothenburg, Box- 480, 413 45 Gothenburg, Sweden; 2 Department of Internal Medicine and Clinical Nutrition, Sahlgrenska Osteoporosis Center, Centre for Bone and Arthritis Research, Institute of Medicine, Sahlgrenska Academy, University of Gothenburg, Gothenburg, Sweden; 3 Centre for Clinical Laboratories, The Affiliated Hospital of Guizhou Medical University, Guiyang, China; 4 Department of Rheumatology, Sahlgrenska University Hospital, Gothenburg, Sweden; 5 SciLifeLab, University of Gothenburg, Box 413, 405 30 Gothenburg, Sweden

**Keywords:** Adaptive immunity, Infectious diseases, Infection

## Abstract

Aging alters immunoglobulin production, affecting the humoral immune response. Toll-like receptor 2 (TLR2) recognizes *Staphylococcus aureus* (*S. aureus*) which causes bacteremia with high mortality in the elderly. To understand how TLR2 and aging affect the humoral immune response in bacteremia, four groups of mice (wild type-young, wild type-old, TLR2^−/−^-young, and TLR2^−/−^-old) were used to analyze immunoglobulin levels in healthy conditions as well as 10 days after intravenous injection with *S. aureus*. We found that aging increased the levels of both IgM and IgG. Increased IgG in aged mice was controlled by TLR2. In bacteremia infection, aged mice failed to mount proper IgM response in both wild-type (WT) and TLR2^−/−^ mice, whereas IgG response was impaired in both aged and TLR2^−/−^ mice. Aged mice displayed reduced IgG1 and IgG2a response irrespective of TLR2 expression. However, impaired IgG2b response was only found in aged WT mice and not in TLR2^−/−^ mice. Both aging and TLR2^−/−^ increased the levels of anti-staphylococcal IgM in bacteremia. Aging increased sialylated IgG in WT mice but not in TLR2^−/−^ mice. IgG sialylation was not affected by the infection in neither of the mice. In summary, aging increases all immunoglobulins except IgG1. However, aged mice fail to mount a proper antibody response to *S. aureus* bacteremia. TLR2 plays the regulatory role in IgG but not IgM response to infection.

## Introduction

The humoral immune system, mediated by immunoglobulins (Ig’s) secreted by B cells or plasma cells, is an essential part of the defense mechanism^1^. Immunoglobulins are classified into five subclasses IgA, IgG, IgM, IgD, and IgE^1,2^. IgM is the largest and the first antibody to appear in the response to initial exposure to antigen and works as a primary barrier against pathogens. IgM binds to the complement system and activates the classical pathway, leading to antigen opsonization. IgG is the most  secreted and one of the most abundant Ig proteins in serum accounting for approximately 10–20% of all plasma proteins^2^. IgG is divided into subclasses IgG1, IgG2a, and IgG2b^2,3^. IgG effector functions include neutralization, complement activation, and regulation of the immune cells where the constant fragment crystallizable (Fc) part of IgG interacts with Fcγ receptors (FcγRs)^3^. There are four classes of FcγRs, where I, III, and IV activate the immune action while IIb inhibits immune action^4^. The IgG subclasses differ in their ability to mediate effector function, where IgG1 shows the highest affinities to the inhibitory FcγR, FcγRIIB and cannot activate the complement system^3^. IgG2a has a higher affinity for activating FcγRs, which can trigger more potent immune responses, while IgG2b has higher affinity with inhibitory FcγRs, which can dampen immune responses. Therefore, IgG2a is generally considered to be a more effective antibody in promoting immunity, whereas IgG2b is considered to be more involved in regulating immune and the complement systems^5^. Independent of the subclasses of IgG the interaction capability to FcγRs is directly regulated by attached glycans on the Fc fragment of IgG conserved at the asparagine 297 position and these glycans structures on antibodies are terminated by sialic acid. The presence of glycans on the Fc fragment impacts the binding affinity to FcγRs, making IgG less potent and the presence of sialic acid on the terminal glycan chain has been shown to shift the IgG to an anti-inflammatory rather than a pro-inflammatory environment. Changes in IgG glycosylation patterns have been linked to a variety of physiological states and diseases, including aging and age-related diseases^1,6^.

Bacteremia is a bacterial infection occurring in the bloodstream. A bacteremia may develop into sepsis and septic shock, causing overwhelming inflammation, immune system dysfunction, multiple organ failure, and death^7–9^. Gram-positive bacteria *Staphylococcus aureus* (*S. aureus*) is one of the most common pathogens that cause various infections in hospitals and communities, posing a medical challenge^10,11^. The surface protein A (SpA) of *S. aureus* not only interacts with the Fab part but shows a direct interaction with Fc part of the IgG^12^. SpA has a complex structure with several immunoglobulin binding sites^13^. *S. aureus* bacteremia displays higher mortalities than bacteremia caused by most other microbes^8,14^. Toll-like receptor 2 (TLR2) recognizes staphylococcal lipoproteins that are one of the pathogens associated molecular patterns on *S. aureus* and play a potent role in the pathogenesis of staphylococcal infections^15^. TLR2 is primarily found in innate cells but it is also found in B cells and plasma cells^16,17^. TLR2’s role in the humoral response to bacterial antigens as well as its mechanism of action is still largely unknown. Aging is known to affect both the quantitative and qualitative aspects of the humoral immune response, changing the specificity and class of antibodies produced^18–20^. The change in the humoral response during aging contributes significantly to the elderly's susceptibility to infectious disease and reduces the protective effects of treatment or vaccination. In using the same experimental setting, Hu et al.; 2023, demonstrated that both aging and TLR2 deficiency enhanced bacteremia^14^. Aging and TLR deficiency enhanced disease susceptibility, but only aging increased mortality. On the other hand, only TLR deficiency affected weight loss, bacterial load, and increased bacterial count in the kidney. So far, the regulation of humoral immune response underlying the age-related, TLR2 and *S. aureus* bacteremia remains largely unknown. In this study, we investigated the effects of TLR2 and aging on the humoral immune response to *S. aureus* bacteremia in a murine model. Our finding shows that in general humoral immune responses to *S. aureus* bacteremia are regulated by both aging and TLR2. Interestingly, the responses of immunoglobulin subclasses to infection are controlled by aging and TLR2 in distinct ways.

## Results

### IgM response to *S. aureus* bacteremia is totally abolished in aged mice irrespective of TLR2 expression

**Figure 1:**
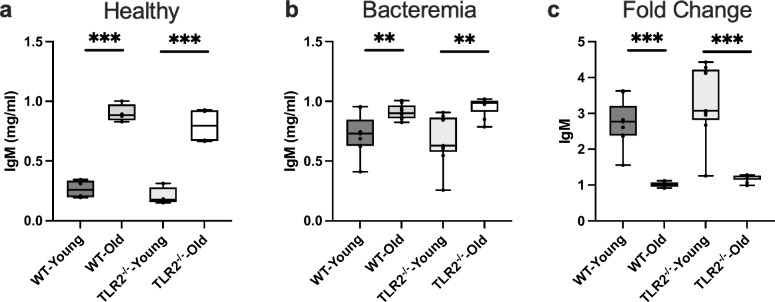
IgM response to infection is totally abolished in aged mice irrespective of TLR2 expression: C57BL/6 wild-type (WT) mice and Toll-like receptor 2-deficient (TLR2^−/−^) mice of both sexes, aged from 13–28 weeks (young) and 73–89 weeks (old). (**a**) IgM levels (mg/ml) in healthy condition (WT-young n = 4, WT-old n = 4, TLR2^−/−^-young n = 4, and TLR2^−/−^-old n = 4). (**b**) IgM levels (mg/ml) at day 10 post-infection of *S. aureus* Newman strain at a dose of 1.5 × 10^6^ CFU/mouse, bacteremia infection, (WT-young n = 8, WT-old n = 9, TLR2^−/−^-young n = 9, and TLR2^−/−^-old n = 9). (**c**) Fold change of IgM levels in bacteremia infection compared to healthy conditions. Statistical evaluations were performed using the student *t* test. Data are presented as box plots and whiskers. **P < 0.01, ***P < 0.001. The data shown are a combination of two individual experiments.

The humoral immune response has been observed to vary in the elderly population compared to young individuals. To understand humoral immunity changes concerning age in healthy WT and TLR2^−/−^ mice, we characterized serum immunoglobulin levels. IgM levels were significantly elevated in both WT and TLR2^−/−^ aged mice compared with their young counterparts (Fig. 1a). After infection, aged mice still had higher levels of IgM compared to the young mice (Fig. 1b). However, comparing the response to the infection, IgM levels were only elevated in the young mice and remained unchanged in aged mice (Fig. 1c). Importantly, IgM levels exhibited approximately a threefold induction after infection in both WT and TLR2^−/−^ young mice. However, IgM levels after infection were the same as before infection in aged mice irrespective of TLR2 expression.

### IgG response to *S. aureus* bacteremia is age- and TLR2-dependent

**Figure 2:**
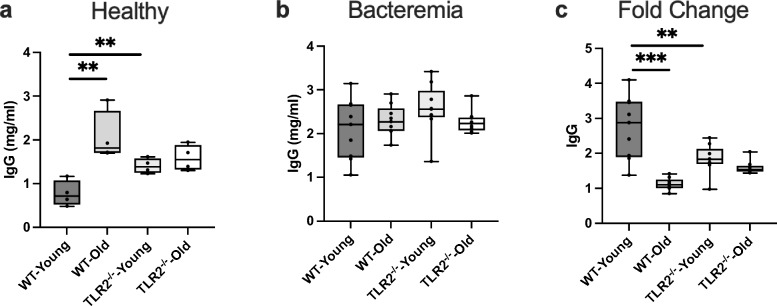
IgG response to *S. aureus* bacteremia is age- and TLR2-dependent: C57BL/6 wild-type (WT) mice and Toll-like receptor 2-deficient (TLR2^−/−^) mice of both sexes, aged from 13–28 weeks (young) and 73–89 weeks (old). (**a**) IgG levels (mg/ml) in healthy condition (WT-young n = 4, WT-old n = 4, TLR2^−/−^-young n = 4, and TLR2^−/−^-old n = 4). (**b**) IgG levels (mg/ml) at day 10 post-infection of *S. aureus* Newman strain at a dose of 1.5 × 10^6^ CFU/mouse, bacteremia infection, (WT-young n = 8, WT-old n = 9, TLR2^−/−^-young n = 9, and TLR2^−/−^-old n = 9). (**c**) Fold change of IgG levels in bacteremia infection compared to healthy conditions. Statistical evaluations were performed using the student *t* test. Data are presented as box plots and whiskers. **P < 0.01, ***P < 0.001. The data shown are a combination of two individual experiments.

Before infection, IgG levels were significantly elevated in healthy WT-aged mice compared to WT-young mice, whereas IgG levels did not change with age in TLR2^−/−^ mice (Fig. 2a). The IgG levels were significantly increased in TLR2^−/−^ young mice compared to WT young mice (Fig. 2a). This data indicates that IgG production is both age- and TLR2-dependent in healthy individuals. Following *S. aureus* infection, similar levels of IgG were observed in all four groups (Fig. 2b). We further estimated the IgG response to infection by calculating the fold changes of IgG levels after infection compared with the levels before infection. In WT young mice IgG levels were increased around three times after infection, but no significant increase was visible in either aged WT or TLR2^−/−^ mice (Fig. 2c). This suggests that the IgG response to infection is affected by both age and TLR2.

### The impact of aging and TLR2 on IgG subclasses in healthy and *S. aureus* bacteremia

**Figure 3:**
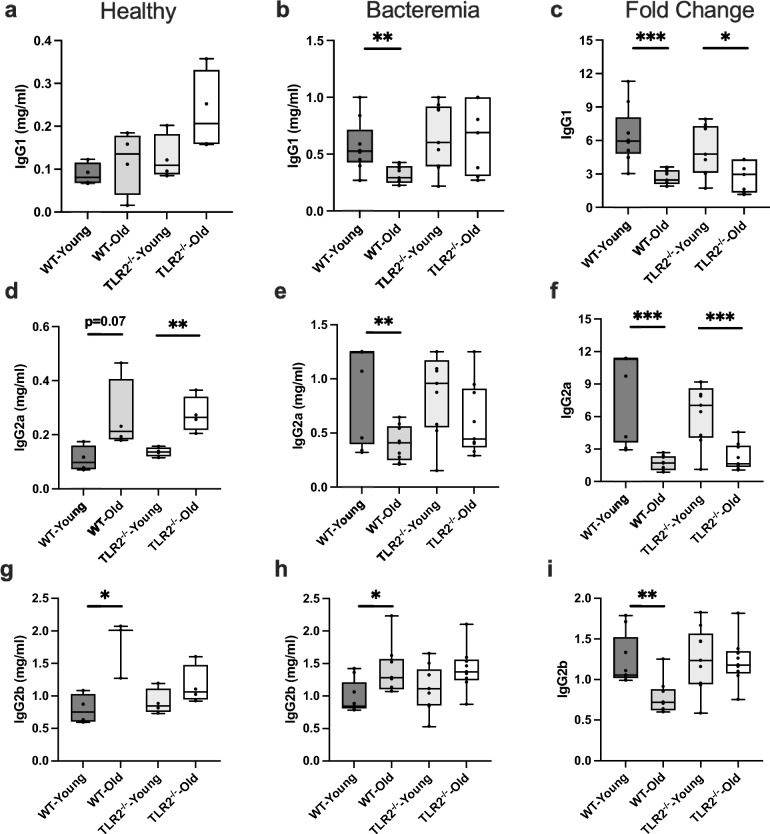
The impact of aging and TLR2 on IgG subclasses in healthy and *S. aureus* bacteremia: C57BL/6 wild-type (WT) mice and Toll-like receptor 2-deficient (TLR2^−/−^) mice of both sexes, aged from 13–28 weeks (young) and 73–89 weeks (old). (**a**) IgG1 levels (mg/ml) in healthy condition (WT-young n = 4, WT-old n = 4, TLR2^−/−^-young n = 4, and TLR2^−/−^-old n = 4). (**b**) IgG1 levels (mg/ml) at day 10 post-infection of *S. aureus* Newman strain at a dose of 1.5 × 10^6^ CFU/mouse, bacteremia infection, (WT-young n = 8, WT-old n = 9, TLR2^−/−^-young n = 9, and TLR2^−/−^-old n = 9). (**c**) Fold change of IgG1 levels in bacteremia infection compared to healthy conditions. (**d**) IgG2a levels (mg/ml) in healthy conditions. (**e**) IgG2a levels (mg/ml) in bacteremia infection. (**f**) Fold change of IgG2a levels in bacteremia infection compared to healthy conditions. (**g**) IgG2b levels (mg/ml) in healthy condition. (**h**) IgG2b levels (mg/ml) in bacteremia infection. (**i**) Fold change of IgG2b levels in bacteremia infection compared to healthy conditions. Statistical evaluations were performed using the student *t* test Data are presented as box plots and whiskers. *P < 0.05, **P < 0.01, ***P < 0.001. The data shown are a combination of two individual experiments.

To understand the relationship between different IgG subclasses with age and TLR2 under healthy- and the infectious condition, we further analyzed different IgG subtypes. IgG1 was not affected by either age or lack of TLR2 in healthy mice (Fig. 3a). After infection, IgG1 levels were significantly decreased in aged WT mice compared with their WT-young counterpart (Fig. 3b). However, no difference was observed depending on age in TLR2^−/−^ mice (Fig. 3b). The fold changes of IgG1 response increased in all four groups and aging significantly decreased IgG1 response in both WT and TLR2^−/−^ mice (Fig. 3c). In healthy conditions, age increased IgG2a levels in both WT and TLR2^−/−^ mice (Fig. 3d). After infection, the levels of IgG2a increased more than two-fold in WT mice compared to healthy conditions, although aged WT mice showed a lower level of IgG2a compared to the WT-young (Fig. 3e). However, in both young and old TLR2^−/−^ mice the IgG2a levels increased compared to healthy, and slight reduction in the aged mice (Fig. 3e). When calculating the fold change, IgG2a response to *S. aureus* infection was significantly dampened by aging in both aged WT and TLR2^−/−^ mice (Fig. 3f). IgG2b which is the most dominant subtype of IgG that followed the total IgG response as shown in (Fig. 2a), we found that in healthy conditions, IgG2b levels in WT-aged mice significantly increased compared to their young counterparts but did not change in the TLR2^−/−^ mice (Fig. 3g). The same pattern was also visualized in infection, with an increased level of IgG2b in WT-aged mice compared to WT-young (Fig. 3h). Fold change with respect to infection, the level of IgG2b was significantly decreased by aging in WT mice but not in TLR2^−/−^ mice (Fig. 3i). Our data indicate that the different subtypes of IgG are tightly regulated by all three factors, age, TLR2, and infection in distinct patterns.

### *S. aureus-specific* anti-IgM but not anti-IgG are dependent on age and TLR2

**Figure 4:**
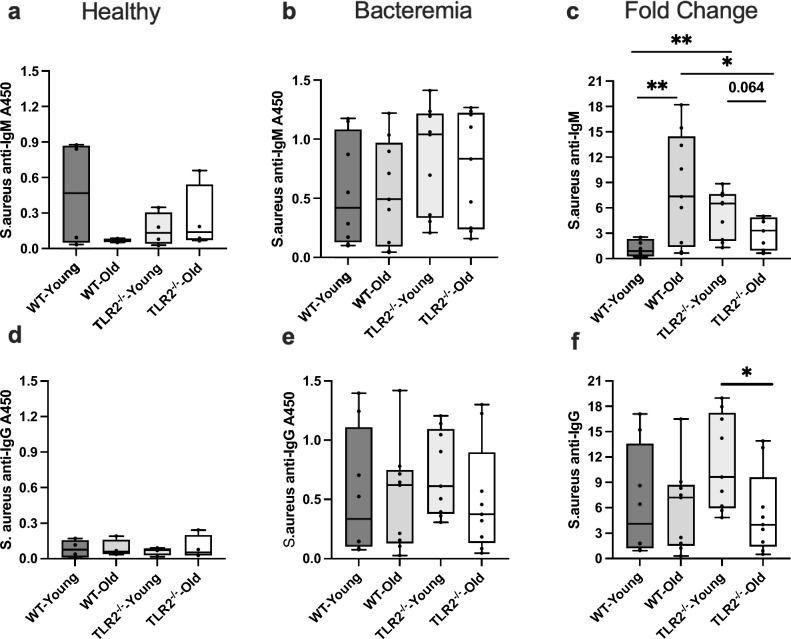
*S. aureus*-specific anti-IgM but not anti-IgG levels are dependent on age and TLR2: C57BL/6 wild-type (WT) mice and Toll-like receptor 2-deficient (TLR2^−/−^) mice of both sexes, aged from 13–28 weeks (young) and 73–89 weeks (old). (**a**) *S. aureus*-specific anti-IgM levels (A450) in healthy conditions (WT-young n = 4, WT-old n = 4, TLR2^−/−^-young n = 4, and TLR2^−/−^-old n = 4). (**b**) *S. aureus*-specific anti-IgM levels (A450) at day 10 post-infection of *S. aureus* Newman strain at a dose of 1.5 × 10^6^ CFU/mouse, bacteremia infection, (WT-young n = 8, WT-old n = 9, TLR2^−/−^-young n = 9, and TLR2^−/−^-old n = 9). (**c**) Fold change of *S. aureus*-specific anti-IgM levels in bacteremia infection compared to healthy conditions. (**d**) *S. aureus*-specific anti-IgG levels (A450) in healthy conditions. (**e**) *S. aureus*-specific anti-IgG levels (A450) in bacteremia infection. (**f**) Fold change of *S. aureus*-specific anti-IgG levels in bacteremia infection compared to healthy conditions. Statistical evaluations were performed using the student *t* test Data are presented as box plots and whiskers. *P < 0.05, **P < 0.01. The data shown are a combination of two individual experiments.

It has previously been reported that serum levels of antibodies recognizing *S. aureus* components can be measured not only in infected patients but also in clinically healthy individuals^10^. The levels of *S. aureus*-specific IgM were detectable in healthy mice with no alteration depending on age or TLR2^−/−^ (Fig. 4a). After *S. aureus-induced* bacterium, we found an increase in antigen-specific IgM levels in all mice, indicating that the levels were increased due to infection (Fig. 4b). In all mice, the fold changes in *S. aureus*-specific IgM response were dramatically increased more than two-fold (Fig. 4c). The levels of *S. aureus*-specific IgM were significantly higher in WT-aged and TLR2^−/−^ young mice compared to WT-young mice. TLR2^−/−^ old mice also displayed significantly lower antigen-specific IgM levels compared to WT-old and a strong trend compared to TLR2^−/−^ young mice. This suggests that antigen-specific IgM is both age- and TLR2-dependent. In healthy conditions, the levels of *S. aureus*-specific IgG were detectable in all mice (Fig. 4d). Following the *S. aureus*-induced bacterium, the levels of *S. aureus*-specific IgG increased independent of age or TLR2^−/−^ (Fig. 4e). When calculating the fold change, *S. aureus*-specific IgG levels were more than six-fold higher in the bacterium condition (Fig. 4f). Similar induction was displayed in all groups with only a decrease in TLR2^−/−^ old mice compared to their younger counterpart suggesting that antigen-specific IgG is influenced by TLR2 in bacterium conditions.

### Sialylated IgG levels are dependent on age and TLR2 rather than *S. aureus* bacteremia

**Figure 5:**
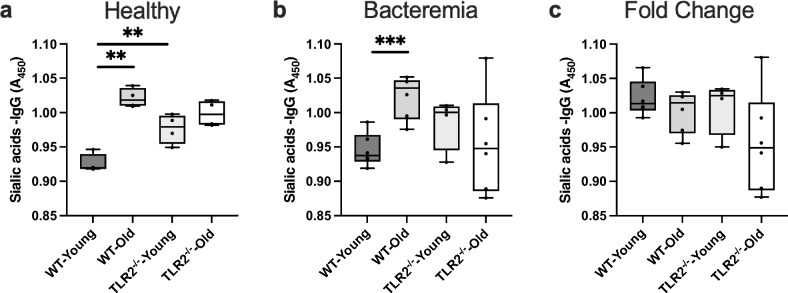
Sialylated IgG levels are dependent on age and TLR2 rather than *S. aureus* bacteremia: C57BL/6 wild-type (WT) mice and Toll-like receptor 2-deficient (TLR2^−/−^) mice of both sexes, aged from 13–28 weeks (young) and 73–89 weeks (old). (**a**) Sialic acid on IgG in healthy condition (WT-young n = 4, WT-old n = 4, TLR2^−/−^-young n = 4, and TLR2^−/−^-old n = 4). (**b**) Sialic acid on IgG at day 10 post-infection of *S. aureus* Newman strain at a dose of 1.5 × 10^6^ CFU/mouse, bacteremia infection, (WT-young n = 8, WT-old n = 9, TLR2^−/−^-young n = 9, and TLR2^−/−^-old n = 9). (**c**) Fold change Sialic acid on IgG levels in bacteremia infection compared to healthy conditions. Statistical evaluations were performed using the student *t* test. Data are presented as box plots and whiskers. **P < 0.01, ***P < 0.001. The data shown are a combination of two individual experiments.

The degree of glycosylation in IgG influences the binding capacity to the FcγR and thereby its effector functions. We examined whether TLR2 and aging affect the sialic acid levels on IgG. We found that in healthy WT-aged mice, the sialic acid levels on IgG increased compared to WT-young mice (Fig. 5a). Also, TLR2^−/−^ young mice showed an increment of sialic acid levels on IgG compared to WT-young mice. Intriguingly, there was no difference in the TLR2^−/−^ mice concerning age, suggesting the regulatory role of TLR2 in IgG sialylation during aging. In infection, a similar pattern was observed as we found in the healthy condition an increased level of sialic acid on IgG in WT-aged mice compared to WT-young mice (Fig. 5b). Similar to healthy, we did not find any alteration based on age in TLR2^−/−^ mice after the infection. Finally, looking at the fold change with respect to infection, we did not see any change in the levels of sialic acid on IgG depending on age and TLR2 (Fig. 5c).

### T- and B-cells are only TLR2 while plasma cells are both age and TLR2-dependent in healthy conditions

Next, we characterized the development of lymphoid tissue-derived T-, B-, and plasma cells in mice under healthy conditions. The frequency of total T-cells did not change with age, although it was significantly higher in TLR2^−/−^ young mice compared to WT-young mice (Supplementary Table 1). B-cell frequency remains unaltered in both WT and TLR2^−/−^ mice in young compared to old mice. On the other hand, TLR2^−/−^ young mice had significantly lower B-cells than WT-young mice. Further, we analyzed the plasma cell population and found an increased frequency in both aged WT and TLR2^−/−^ mice compared to their younger counterparts. This alteration was more visible in WT mice compared to TLR2^−/−^ mice (Supplementary Table 1). These findings suggest that T- and B-cells are TLR2-dependent rather than age, whereas plasma cells are both TLR2 and age-dependent.

## Discussion

Alterations in the humoral immune response are related to immunoglobulins concentration, changes in the antibody affinity as well as the number and activity of B cells^21^. The aging process is complex, with changes in both innate and adaptive immunity leading to increased susceptibility to infections and decreasing ability to protect itself^22^. Both antibody-mediated and T-cell-mediated cellular immune responses have previously been shown to decline with age in both humans and experimental animals^23,24^, contributing significantly to the elderly's increased susceptibility to infections with reduced protective effects of antigen stimulation^25,26^. During bacterial infections, TLRs such as TLR4 and TLR2 become activated^16^. According to a prior study, TLR2 plays an important role in the synthesis of immunoglobulins by shaping their response to the disease^27^. Indeed, the clinical data from the same experimental setting have demonstrated that age and TLR2 deficiency synergically aggravated the *S. aureus* bacteremia^14^. The mortality and spleen weight changes caused by *S. aureus* bacteremia and are controlled by aging, whereas other clinical parameters such as weight loss and kidney abscesses are TLR2 dependent. Here, we show that both age and TLR2, influence humoral immune responses to *S. aureus* bacteremia. Surprisingly, the responses of IgG subclasses to infection are controlled in different ways by aging and TLR2.

Natural antibodies are formed spontaneously, and are mainly of the IgM class, without any exposure to foreign antigens as part of the first line of defense, whereas antigen-specific antibodies are formed in response to an infectious antigen. The number of natural antibodies declines with age but at the same time, the antigen-specific antibodies increase with age. Aged individuals thereby produced fewer antibodies that are more specific for the activating pathogen but less prone to respond to a new antigen. The TLR's role in regulating the natural contra antigen-specific antibodies is largely unknown.

IgM is the first antibody and mainly a natural antibody that responds to the pathogen’s exposure. In comparison to the young, the IgM response in the elderly appears to be very inconsistent, as reported in previous studies in both humans and animals^21,28–33^. Our current study showed that IgM levels in healthy WT and TLR2^−/−^ aged mice were significantly higher compared to their young counterparts. We found that aged WT and TLR2^−/−^ mice have higher IgM levels after infection than young mice which is consistent with the previous report^34^. It is well known that reduced IgM memory B cells in the elderly result in lower IgM titers after immunization against pneumococcal polysaccharide and streptococcal pneumonia infections^35^. Herein we demonstrated the IgM response to infection was dramatically induced in young mice in both genotypes compared with their counterparts, whereas no major alteration in aged mice. The lack of IgM induction in bacteremia in the aged mice may be related to the lack of IgM B-cell proliferation in elderly individuals, as previously studied in^21^.

*Radl *et al. showed that aged individuals with no infection have higher IgG titers compared to the younger population^32^. Furthermore, IgG produced by old mice and humans is less protective than IgG produced by young individuals^18–20^. IgG levels reach their peak in healthy youth between 13 to 15 years of age and then begin to decline at 16 years of age and onwards, implying that most antibodies are formed during childhood^10^. In the present study, we demonstrated that IgG levels in healthy aged WT mice were significantly higher compared to young WT mice, whereas IgG levels in TLR2^−/−^ mice did not change with age. IgG levels were also slightly increased in young TLR2^−/−^ mice compared to young WT mice. It is known that specific IgG responses decline with age in humans vaccinated against various bacterial and influenza strains^10,29,36,37^. TLR2 has previously been shown to shape the IgG antibody response to bacterial infection^16^. In oral immunization of cholera toxin in an experimental setup the total serum IgG did not change with age^31^. Like the previous studies, after bacteremia infection, we found that in *S. aureus* infected mice the total IgG levels were similar in all mice and neither affected by age nor the lack of TLR2. Indeed, when we compared the response in the healthy towards the bacteremia infection the level of IgG was three times higher in WT-young mice, with only a minor induction in TLR2^−/−^ mice and no alteration in old WT mice. This shows that the aged WT and partially TLR2^−/−^ mice cannot further increase IgG in response to bacteremia infection, which may be important for less immune clearance in both aged and TLR2 deficient mice.

Previously it has been reported that serum IgG subclass levels were decreased in aged nude mice compared to younger mice^38^ whereas these IgG subclasses remained fundamentally unchanged in healthy individuals aged between 24–98^39^. Here, we found that IgG1 levels were neither altered by age nor by TLR2 deficiency while age increased IgG2a levels in both WT and TLR2^−/−^ mice in healthy conditions. IgG2b, the most prevalent form of IgG that follows the total IgG response, increased significantly in aged WT mice compared to their young counterparts but did not change in TLR2^−/−^ mice. In a murine study of polymicrobial abdominal sepsis, serum IgG1, and IgG2b concentrations are increased during the course of sepsis^40^. Post-immunization analysis of bacterial infection and in the *Ischemia* mode*l*, IgG subclasses in TLR2^−/−^ mice showed a minor increase in IgG1 and IgG2b titers, but a significant decrease in IgG2a titers^16,27^. Interferon-gamma (IFN-γ) is a cytokine that has an important physiological role in altering the subtypes of IgG, with increased production of IgG2a as well as a decreased production of IgG1 during LPS activation of B-lymphocytes^41^. We found that after bacteremia infection, IgG1 and IgG2a levels were significantly decreased in aged WT mice compared with their young counterpart, but IgG2b showed a significant increase in aged WT mice. However, IgG subclasses were not altered after bacteremia infection in either young or aged TLR2^−/−^ mice. The reduced IgG2a response to *S. aureus* infection in both WT and TLR2^−/−^ mice could be due to FcγRs activation, affecting the binding affinity^42^. Here we observed that both IgG1 and IgG2a levels were increased in all mice in response to the infection, but these responses were more induced in both young WT and TLR2^−/−^ mice than in their aged counterparts. When comparing infection versus healthy mice, aging significantly reduced IgG2b response in WT mice but not in the TLR2 deficient mice. This implies that only IgG1 and IgG2a levels increased due to the bacteremia infection but in a higher proportion in the young versus the aged in both WT and TLR2^−/−^ mice. The IgG2b, the most prevalent form, was not altered by infection but higher levels were visible in old WT compared to their younger counterparts in both healthy and infectious conditions. The lower IgG2b response observed in aged WT mice against infection, here in this study may be related to the lower total IgG titer in aged mice against bacteremia infection as in (Fig. 2C).

In oral immunization of cholera toxin, an experimental setup the antigen-specific IgG levels decline in aged mice^31^. In contrast to this previous study, we found no variation in the levels of antigen-specific immunoglobulins against SpA with respect to age or TLR2 in both healthy and infected mice. Although infected mice displayed a higher range of both SpA-specific IgM and IgG levels than the healthy mice. Indicating that a large proportion of the SpA-specific IgM could be natural antibodies.

IgG glycans are one of the major factors known to accelerate the aging process by modulating the immune activation threshold^6,43–45^. The fact that certain sugar moiety residues influence both the pro- and anti-inflammatory activity of the IgG has come as a big surprise in recent years^44^. Previously it has been shown that the IgG sialylation does not generally change with age^45^. However, one study showed that individuals over the age of 65 have less sialic acid in their IgG^43^. Here we demonstrated that aging increases the sialic acid on IgG in aged WT mice compared to their young counterparts in both healthy and bacteremia mice. In addition, TLR2^−/−^ young mice have higher levels of sialic acids on IgG than the WT-young mice in healthy conditions. Interestingly, no difference was found in TLR2^−/−^ mice concerning age in either healthy or infected mice, suggesting that TLR2 may play a regulatory role in IgG sialylation during aging.

Aging affects B-cell response quantitatively by reducing antibody production^19^ which is due to an overall decline in the bone marrow output and a decrease in the number of hematopoietic stem cells during the T- and B-cell development, resulting in decreasing number of T- and B-cells in the elderly as reviewed previously in^46^. In contrast to the previous reports, we found no difference in the frequency of T- and B-cell depending on age. TLR2^−/−^ young mice, on the other hand, show a significant stimulatory effect on both T- and B-cell numbers implying that TLR2 plays an important role in T- and B-cell development. It is well-known from the literature, that plasma cell production increases with age, and we have also shown that both old WT and TLR2^−/−^ mice had significantly higher numbers compared to their younger counterparts^19^. This increase was more evident in aged WT mice compared to aged TLR2^−/−^ mice, indicating that TLR2 plays a significant role in plasma cell regulation, as previously described in^47^.

**Figure 6:**
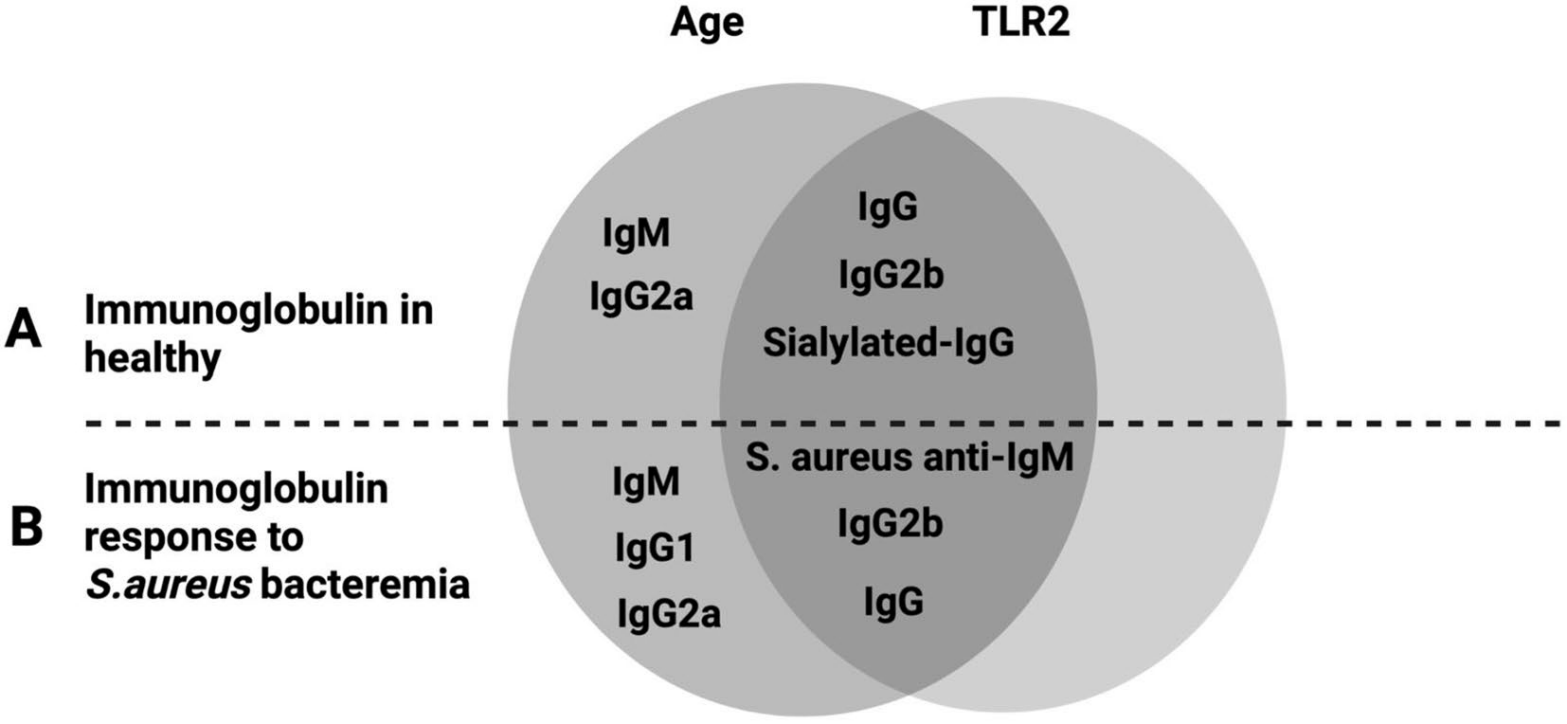
Result summary: Immunoglobulins response concerning age and TLR2: (**A**) healthy, and (**B**) fold changes of immunoglobulins response for age and TLR2 to *S. aureus* bacteremia in comparison to respective groups of healthy mice.

Our results show that age but not TLR2 tightly controls the IgM production in both healthy conditions as well as in responses to *S. aureus* bacteremia (Fig. 6). In contrast, both age and TLR2 regulate the IgG and its subclasses, especially IgG2b production in healthy mice and IgG2b responses to infection as well as antigen-specific IgM levels. In summary, our data demonstrate a subtle relationship between humoral immune response, aging, and TLR2 in *S. aureus*-induced bacteremia.

## Materials and methods

### Ethics statement

Mouse experiments were conducted in accordance with recommendations listed in the Swedish Board of Agriculture’s regulations and recommendations on animal experiments. Mouse studies were reviewed and approved by the Ethics Committee of Animal Research of Gothenburg. This study is reported in accordance with ARRIVE guidelines.

### Mice

C57BL/6 wild-type mice and Toll-like receptor 2-deficient B6.129-Tlr2^tm1Kir^/J (TLR2^−/−^) mice were purchased from The Jackson Laboratory (Bar Harbor, Maine, USA). Mice were housed and bred in the animal facility under standard conditions until they reached the required age range as young (13–28 weeks old) and old mice (73–89 weeks old), respectively. The Ethics Committee of Animal Research of Gothenburg approved the study, and the animal experimentation guidelines were strictly followed.

### Preparation of bacterial solutions

*S. aureus* Newman wild-type strain was prepared as previously described in^48^. Briefly, premade batches of bacterial strain were stored at − 20 ℃ in a phosphate buffer saline-based freezing solution, containing 5% bovine serum and 10% dimethyl sulfoxide until use.

### Experimental protocol for hematogenous bacteremia mouse model

A mouse bacteremia model was performed as previously described by *Kwiecinski, J., et al*^49^. Briefly, 200 µl of *S. aureus* suspension (1.5 × 10^6^ CFU/mouse) was injected intravenously into the tail vein of mice. The animals were monitored daily for up to 10 days after infection. The data were pooled from two independent experiments. All 4 groups of mice were included in each experiment to make wild type-young (n = 8), wild type-old (n = 9), TLR2^−/−^-young (n = 9), and TLR2^−/−^-old (n = 9).

### Sample collection

On termination at day 10, the blood sample was collected into tubes containing EDTA followed by centrifugation at 10,000 rpm for 10 min at RT.

### *S. aureus* antigen preparation from culture supernatant

The culture supernatant fraction was prepared by ethanol precipitation as previously described in^10^. Briefly, culture supernatants of *S. aureus* ΔSpA strain (protein A deficient strain) cultures were grown overnight in Tryptic Soy Broth (TSB) medium, were incubated with 3 volumes of ice-cold 90% ethanol at − 20 °C for at least overnight, and precipitated proteins were collected by centrifugation at 10,000 rpm for 10 min at 4 °C. The pellet was dried and resuspended in PBS. Protein concentrations were determined by a commercially available DCA protein assay kit (DCA protein assay; Bio-Rad, Sweden).

### ELISA analyses

Total serum IgM and IgG levels were measured using a commercially available kit (Bethyl Laboratories, E90-10, and E90-13 respectively, Nordic Biosite, Sweden). Specific IgG isotypes IgG1, IgG2a, and IgG2b in serum were measured using commercially available ELISA kits (Thermo fisher, 88-50410, 88-50420, and 88-50430 respectively, Sweden) according to the manufacturer’s protocol. The sialic acids on IgG were measured using an in-house Lectin ELISA, as described previously in^50^. Briefly, anti-mouse IgG F(ab)2 fragment (Sigma Aldrich, M0659, Sweden) was used to coat 96 well plates overnight at 4 °C in sodium bicarbonate buffer (0.1 M, pH 9.6), then blocked for 1–2 h using polyvinyl pyrrolidone (0.5%) (Sigma Aldrich, Sweden) in warm TBS. Incubated with serum in TBST (0.1% tween 20) followed by biotinylated Sambucus nigra lectin (SNA) to detect the sialic acid (1:4000 dilution) (Vector Laboratories, B-1305-2, Sweden) on IgG, followed by streptavidin-HRP (R&D Systems, DY998, Sweden) were used to detect sialylation. *S. aureus* antigen-specific IgG and IgM ELISA were performed according to standard protocols. Briefly, 96-well plates (Maxisorp; NUNC, Fisher Scientific, Sweden) were coated with antigens (100 μl) diluted in 0.1 M NaHCO_3_ buffer (pH 9.3) to a concentration of 10 μg/ml for culture supernatant proteins prepared from an *S. aureus* strain. The plates were then incubated overnight at 4 °C, blocked with 1% Bovine serum albumin (BSA)-PBS buffer for 1–2 h, followed by mice sera (1:1000) incubation at 1 h at RT. After washing with 0.1% PBS-T, HRP conjugated anti-mouse antibodies for IgG and IgM detection (Bethyl lab. Nordic Biosite, Sweden) in 1:1000 dilution was added to each well and incubated for 1 h at RT. OD was measured at 450 nm using a microplate reader.

### Flow cytometry analyses

Single-cell suspension was prepared from the spleen in PBS for flow cytometry analyses. Cells were counted using an automated cell counter (Sysmex Europe GmBH, Sweden). Flow cytometry analysis was performed using different fluorochrome-conjugated antibodies i.e. APC-anti-CD267 (TACI), PerCP-anti-CD19, BV421-anti-CD138, V500-anti-B220, and APC-Cy7-anti-CD3, all purchased from eBioscience (Sweden). Analysis was performed using the BD FACS-verse flow cytometer and Flow Jo software (FlowJo10.6.2).

### Statistical analyses

Statistical analyses were performed using GraphPad Prism version 9 (GraphPad Software, La Jolla California, USA). Grubb’s test was used to detect outliers and consequently excluded from the analysis. Statistical evaluations were performed using the student T-test and compared to the age group within each mouse strain (young vs old) as well as between the WT and KO mice (young vs young and old vs old). Data are presented as box plots and whiskers and a *P*-value < 0.05 was considered statistically significant.

## Supplementary Information

Supplementary Information.

## Data Availability

The data supporting this study's findings are openly available in the figshare repository, https://doi.org/10.6084/m9.figshare.22200001.

## References

[1] Gudelj I, Lauc G, Pezer M (2018). Immunoglobulin G glycosylation in aging and diseases. Cell. Immunol..

[2] Vidarsson G, Dekkers G, Rispens T (2014). IgG subclasses and allotypes: From structure to effector functions. Front. Immunol..

[3] Lilienthal GM, Rahmöller J, Petry J, Bartsch YC, Leliavski A, Ehlers M (2018). Potential of murine IgG1 and human IgG4 to inhibit the classical complement and Fcγ receptor activation pathways. Front. Immunol..

[4] Nimmerjahn F, Ravetch JV (2008). Fcγ receptors as regulators of immune responses. Nat. Rev. Immunol..

[5] Buhre JS, Becker M, Ehlers M (2022). IgG subclass and Fc glycosylation shifts are linked to the transition from pre- to inflammatory autoimmune conditions. Front. Immunol..

[6] Krištić J, Lauc G, Pezer M (2022). Immunoglobulin G glycans—Biomarkers and molecular effectors of aging. Clin Chim Acta..

[7] Heindel DW, Chen S, Aziz PV, Chung JY, Marth JD, Mahal LK (2022). Glycomic analysis reveals a conserved response to bacterial sepsis induced by different bacterial pathogens. ACS Infect. Dis..

[8] Jin T, Mohammad M, Pullerits R, Ali A (2021). Bacteria and host interplay in Staphylococcus aureus septic arthritis and sepsis. Pathogens..

[9] Mohammad M, Hu Z, Ali A (2020). The role of Staphylococcus aureus lipoproteins in hematogenous septic arthritis. Sci. Rep..

[10] Dryla A, Prustomersky S, Gelbmann D (2005). Comparison of antibody repertoires against Staphylococcus aureus in healthy individuals and in acutely infected patients. Clin. Diagn. Lab. Immunol..

[11] National Nosocomial Infections Surveillance (NNIS) System Report, data summary from January 1992 to June 2002, issued August 2002. *Am. J. Infect. Control*. 2002;30(8):458–75. 10.1067/mic.2002.130032 10.1067/mic.2002.130032 12461510

[12] Forsgren A, Nordström K (1974). Protein A from Staphylococcus aureus: The biological significance of its reaction with IgG. Ann. N. Y. Acad. Sci..

[13] Falugi F, Kim HK, Missiakas DM, Schneewind O (2013). Role of protein A in the evasion of host adaptive immune responses by Staphylococcus aureus. MBio.

[14] Hu Z, Kopparapu PK, Deshmukh M (2023). The impact of aging and TLR2 deficiency on the clinical outcomes of Staphylococcus aureus bacteremia. J. Infect. Dis..

[15] Mohammad M, Ali A, Nguyen MT, Götz F, Pullerits R, Jin T (2022). Staphylococcus aureus lipoproteins in infectious diseases. Front Microbiol..

[16] Cervantes-Barragán L, Gil-Cruz C, Pastelin-Palacios R (2009). TLR2 and TLR4 signaling shapes specific antibody responses to Salmonella typhi antigens. Eur. J. Immunol..

[17] Nagai Y, Kobayashi T, Motoi Y (2005). The radioprotective 105/MD-1 complex links TLR2 and TLR4/MD-2 in antibody response to microbial membranes. J. Immunol..

[18] Frasca D, Blomberg BB (2009). Effects of aging on B cell function. Curr. Opin. Immunol..

[19] Frasca D, Diaz A, Romero M, Landin AM, Blomberg BB (2011). Age effects on B cells and humoral immunity in humans. Ageing Res. Rev..

[20] Linton PJ, Dorshkind K (2004). Age-related changes in lymphocyte development and function. Nat. Immunol..

[21] Samia Macedo Queiroz Mota Castellao Tavares WdLBJaJLAL. Analyze the levels of immunoglobulins IgG and IgM in elderly and youngs. 2015.

[22] Ginaldi L, Loreto MF, Corsi MP, Modesti M, De Martinis M (2001). Immunosenescence and infectious diseases. Microbes Infect..

[23] Murasko DM, Bernstein ED, Gardner EM (2002). Role of humoral and cell-mediated immunity in protection from influenza disease after immunization of healthy elderly. Exp. Gerontol..

[24] Nicoletti C, Borghesi-Nicoletti C, Yang XH, Schulze DH, Cerny J (1991). Repertoire diversity of antibody response to bacterial antigens in aged mice. II. Phosphorylcholine-antibody in young and aged mice differ in both VH/VL gene repertoire and in specificity. J. Immunol..

[25] McElhaney JE, Effros RB (2009). Immunosenescence: What does it mean to health outcomes in older adults?. Curr. Opin. Immunol..

[26] Steger MM, Maczek C, Berger P, Grubeck-Loebenstein B (1996). Vaccination against tetanus in the elderly: Do recommended vaccination strategies give sufficient protection. Lancet.

[27] Pope MR, Fleming SD (2015). TLR2 modulates antibodies required for intestinal ischemia/reperfusion-induced damage and inflammation. J. Immunol..

[28] Bátory G, Jancsó Á, Puskás É, Rédei A, Lengyel É (1984). Antibody and immunoglobulin levels in aged humans. Arch. Gerontol. Geriatr..

[29] Dietz S, Lautenschläger C, Müller-Werdan U (2017). Serum IgG levels and mortality in patients with severe sepsis and septic shock. Medizinische Klinik Intensivmedizin und Notfallmedizin..

[30] Faria AM, Ficker SM, Speziali E (1998). Aging and immunoglobulin isotype patterns in oral tolerance. Braz. J. Med. Biol. Res..

[31] Koga T, McGhee JR, Kato H, Kato R, Kiyono H, Fujihashi K (2000). Evidence for early aging in the mucosal immune system. J. Immunol..

[32] Radl J, Sepers JM, Skvaril F, Morell A, Hijmans W (1975). Immunoglobulin patterns in humans over 95 years of age. Clin. Exp. Immunol..

[33] Speziali E, Santiago AF, Fernandes RM, Vaz NM, Menezes JS, Faria AMC (2009). Specific immune responses but not basal functions of B and T cells are impaired in aged mice. Cell. Immunol..

[34] Rhinehart-Jones TR, Fortier AH, Elkins KL (1994). Transfer of immunity against lethal murine Francisella infection by specific antibody depends on host gamma interferon and T cells. Infect. Immun..

[35] Shi Y, Yamazaki T, Okubo Y, Uehara Y, Sugane K, Agematsu K (2005). Regulation of aged humoral immune defense against pneumococcal bacteria by IgM memory B cell. J. Immunol..

[36] Gardner EM, Bernstein ED, Dran S (2001). Characterization of antibody responses to annual influenza vaccination over four years in a healthy elderly population. Vaccine..

[37] LeMaoult J, Szabo P, Weksler ME (1997). Effect of age on humoral immunity, selection of the B-cell repertoire and B-cell development. Immunol Rev..

[38] Mink JG, Radl J, van den Berg P, Haaijman JJ, van Zwieten MJ, Benner R (1980). Serum immunoglobulins in nude mice and their heterozygous littermates during ageing. Immunology.

[39] De Greef GE, Van Tol MJ, Van Den Berg JW (1992). Serum immunoglobulin class and IgG subclass levels and the occurrence of homogeneous immunoglobulins during the course of ageing in humans. Mech. Ageing Dev..

[40] Nicolai O, Pötschke C, Schmoeckel K (2020). Antibody production in murine polymicrobial sepsis-kinetics and key players. Front. Immunol..

[41] Finkelman FD, Katona IM, Mosmann TR, Coffman RL (1988). IFN-gamma regulates the isotypes of Ig secreted during in vivo humoral immune responses. J. Immunol..

[42] Collins AM (2016). IgG subclass co-expression brings harmony to the quartet model of murine IgG function. Immunol. Cell Biol..

[43] Keusch J, Levy Y, Shoenfeld Y, Youinou P (1996). Analysis of different glycosylation states in IgG subclasses. Clin. Chim. Acta.

[44] Lux A, Aschermann S, Biburger M, Nimmerjahn F (2010). The pro and anti-inflammatory activities of immunoglobulin G. Ann. Rheum. Dis..

[45] Parekh R, Roitt I, Isenberg D, Dwek R, Rademacher T (1988). Age-related galactosylation of the N-linked oligosaccharides of human serum IgG. J. Exp. Med..

[46] Salam N, Rane S, Das R (2013). T cell ageing: Effects of age on development, survival & function. Indian J. Med. Res..

[47] Dorner M, Brandt S, Tinguely M (2009). Plasma cell toll-like receptor (TLR) expression differs from that of B cells, and plasma cell TLR triggering enhances immunoglobulin production. Immunology.

[48] Kwiecinski J, Jacobsson G, Karlsson M (2013). Staphylokinase promotes the establishment of Staphylococcus aureus skin infections while decreasing disease severity. J. Infect. Dis..

[49] Jarneborn A, Mohammad M, Engdahl C (2020). Tofacitinib treatment aggravates Staphylococcus aureus septic arthritis, but attenuates sepsis and enterotoxin induced shock in mice. Sci. Rep..

[50] Engdahl C, Bondt A, Harre U (2018). Estrogen induces St6gal1 expression and increases IgG sialylation in mice and patients with rheumatoid arthritis: A potential explanation for the increased risk of rheumatoid arthritis in postmenopausal women. Arthritis Res. Ther..

