# Supplementary material for: The impact of TLR2 and aging on the humoral immune response to Staphylococcus aureus bacteremia in mice
Source: Sci Rep. 2023 May 31;13:8850. doi: 10.1038/s41598-023-35970-3 (PMC10232519; doi:10.1038/s41598-023-35970-3)
Supplement: Supplementary file 1 — Supplementary Information. [file 41598_2023_35970_MOESM1_ESM.pdf]

**The impact of TLR2 and aging on the humoral immune response to *Staphylococcus aureus* bacteremia in mice**

Priti Gupta[1,2*], Zhicheng Hu[1,3], Pradeep Kumar Kopparapu[1], Meghshree Deshmukh[1], Tibor Sághy[1,2], Majd Mohammad[1], Tao Jin[1,4#], Cecilia Engdahl[1,2#]

**Supplementary Table**

**Table 1. Frequency of splenocyte-derived T-, B- and plasma cells in naïve condition**

| **Mouse** | **T cells (%)** | | | |
|---|---|---|---|---|
| | **WT-young** | **WT-old** | **TLR2$^{-/-}$-young** | **TLR2$^{-/-}$- old** |
| 1 | 71 | 75.1 | 77.7 | 57.7 |
| 2 | 67.2 | 58.9 | 77.5 | 71.2 |
| 3 | 68.2 | 74.4 | 75.1 | 79.5 |
| 4 | 68.7 | 65 | 75.8 | 77.7 |
| **Mean** | **68.78** | **68.35** | **76.53** | **71.53** |
| **SEM** | **0.80** | **3.90** | **0.64** | **4.94** |
| **p-Value** | | | **0.0003*** | |
| | **B cells (%)** | | | |
| 1 | 48.7 | 41.8 | 33.3 | 38.4 |
| 2 | 48 | 45.6 | 36.7 | 35.3 |
| 3 | 53.1 | 62.7 | 45.6 | 44 |
| 4 | 53 | 59.8 | 46.6 | 59.4 |
| **Mean** | **50.8** | **52.5** | **40.6** | **44.28** |
| **SEM** | **1.42** | **5.16** | **3.28** | **5.35** |
| **p-Value** | | | **0.029*** | |
| | **Plasma cells (%)** | | | |
| 1 | 0.29 | 0.83 | 0.18 | 0.58 |
| 2 | 0.21 | 0.78 | 0.21 | 0.41 |
| 3 | 0.64 | 1.13 | 0.31 | 0.58 |
| 4 | 0.27 | 1.28 | 0.35 | 0.84 |
| **Mean** | **0.35** | **1.01** | **0.26** | **0.60** |
| **SEM** | **0.10** | **0.12** | **0.04** | **0.09** |
| **p-Value** | | **0.006*** | | **0.036$^{\theta}$** <br> **0.013$^{\#}$** |
| *Significant difference compared to *WT-young, $^{\theta}$WT-old, and $^{\#}$TLR2 $^{-/-}$ old* | | | | |
